# Predictive coding with spiking neurons and feedforward gist signaling

**DOI:** 10.3389/fncom.2024.1338280

**Published:** 2024-04-12

**Authors:** Kwangjun Lee, Shirin Dora, Jorge F. Mejias, Sander M. Bohte, Cyriel M. A. Pennartz

**Affiliations:** ^1^Cognitive and Systems Neuroscience Group, Swammerdam Institute for Life Sciences, Faculty of Science, University of Amsterdam, Amsterdam, Netherlands; ^2^Department of Computer Science, School of Science, Loughborough University, Loughborough, United Kingdom; ^3^Machine Learning Group, Centre of Mathematics and Computer Science, Amsterdam, Netherlands

**Keywords:** predictive processing, visual cortex, spiking neural network, Hebbian learning, unsupervised learning, representation learning, recurrent processing, sensory processing

## Abstract

Predictive coding (PC) is an influential theory in neuroscience, which suggests the existence of a cortical architecture that is constantly generating and updating predictive representations of sensory inputs. Owing to its hierarchical and generative nature, PC has inspired many computational models of perception in the literature. However, the biological plausibility of existing models has not been sufficiently explored due to their use of artificial neurons that approximate neural activity with firing rates in the continuous time domain and propagate signals synchronously. Therefore, we developed a spiking neural network for predictive coding (SNN-PC), in which neurons communicate using event-driven and asynchronous spikes. Adopting the hierarchical structure and Hebbian learning algorithms from previous PC neural network models, SNN-PC introduces two novel features: (1) a fast feedforward sweep from the input to higher areas, which generates a spatially reduced and abstract representation of input (i.e., a neural code for the gist of a scene) and provides a neurobiological alternative to an arbitrary choice of priors; and (2) a separation of positive and negative error-computing neurons, which counters the biological implausibility of a bi-directional error neuron with a very high baseline firing rate. After training with the MNIST handwritten digit dataset, SNN-PC developed hierarchical internal representations and was able to reconstruct samples it had not seen during training. SNN-PC suggests biologically plausible mechanisms by which the brain may perform perceptual inference and learning in an unsupervised manner. In addition, it may be used in neuromorphic applications that can utilize its energy-efficient, event-driven, local learning, and parallel information processing nature.

## 1 Introduction

In the midst of chaotic barrages of sensory information, the brain achieves seamless perception of the world. Despite the apparent ease with which the brain achieves such a formidable feat, the problem of perception is computationally difficult, given that the brain has no direct access to the world. This renders perception into an inverse problem (Pizlo, 2001; Spratling, 2017): the brain has to infer a distal stimulus in the physical world (i.e., cause) from proximal sensations coded in the brain (i.e., effect) (Fechner, 1948). Moreover, given inherently noisy and ambiguous sensory information, the problem also becomes ill-posed. For example, an exponentially growing number of object arrangements and viewing conditions in the three-dimensional world can form the same two-dimensional retinal image.

How does the brain overcome such ambiguity, find a unique and stable solution to the inverse problem, and facilitate seamless perception? A confluence of constructivist theories of perception (Helmholtz, 1867; Kant, 1908; MacKay, 1956; Neisser, 1967; Gregory, 1970; Pennartz, 2015) suggests that the brain imposes a priori constraints on possible solutions to the inverse problem based on an internal model of the world shaped by prior knowledge, experience, and context. In light of recent neurophysiological evidence that supports interaction of feedforward sensory inputs and feedback of a priori knowledge (Felleman and Van Essen, 1991; Bastos et al., 2012; Keller et al., 2012; Walsh et al., 2020), predictive coding (PC) has been proposed as a possible neural implementation of perception (Srinivasan et al., 1982; Mumford, 1992; Rao and Ballard, 1999; Friston, 2005; Pennartz et al., 2019). According to PC in its canonical version (Rao and Ballard, 1999), the brain employs hierarchical cortical circuits in which feedback connections carry predictions to lower areas, whereas feedforward connections carry the mismatch between actual and predicted neural activity (i.e., prediction error). The prediction error is used iteratively to correct the internal generative model, allowing to make more accurate inferences, but also to learn from errors. While its computational goal to explain away incoming sensory input resembles the ideas of redundancy reduction from information theory (Shannon, 1948) and the efficient coding hypothesis (Barlow, 1961), the probabilistic formalization of PC algorithms that approximate Bayesian inference (Friston, 2010) builds on the Bayesian brain hypothesis (Knill and Pouget, 2004) and Helmholtz machine (Dayan et al., 1995). In summary, PC offers a Bayes-inspired solution to the inverse and ill-posed problem of perception, and learning thereof, under the imperative of prediction error minimization. A primary goal of PC modeling is therefore to develop perceptual representations, from which inputs can be generatively reconstructed, in a biologically plausible manner, whereas the more “cognitive” goal of stimulus categorization or classification comes in second position.

Thanks to its potential to explain a multitude of cognitive and neural phenomena (Srinivasan et al., 1982; Rao and Ballard, 1999; Hosoya et al., 2005; Jehee et al., 2006; Spratling, 2010, 2016; Huang and Rao, 2011; Wacongne et al., 2012), PC has inspired many theoretical and computational models of perception. On the one hand, there are biologically motivated PC models in the literature to explain neural mechanisms of perception; on the other hand, machine learning inspired models seek missing ingredients that can place the perceptual capacity of artificial intelligence on par with nature's most intelligent machine. However, both approaches lack biological plausibility in their own respect. Biologically motivated PC models demonstrate how PC accounts for neuronal responses such as classical and extra-classical receptive field properties, but whether their efforts can be generalized across the cortical processing hierarchy remains an open question as their models were confined to specific components of the nervous system, such as the retina (Srinivasan et al., 1982; Hosoya et al., 2005), lateral geniculate nucleus (Huang and Rao, 2011), or V1 (Spratling, 2010), or had a limited depth of processing hierarchy (Rao and Ballard, 1999; Spratling, 2010; Wacongne et al., 2012). The machine learning inspired models show remarkable object recognition capabilities but lack the biological plausibility due to their reliance on supervised learning, convolutional filters, and backpropagation of errors (Whittington and Bogacz, 2017; Sacramento et al., 2018; Van den Oord et al., 2018; Wen et al., 2018; Han et al., 2019; Lotter et al., 2020). Meanwhile, there has been an effort to bridge the gap between the two approaches: a deep gated Hebbian PC (Dora et al., 2021) successfully learns internal representations of natural images across multiple layers of the visual processing hierarchy, while exhibiting neuronal response properties such as orientation selectivity, object selectivity, and sparseness. Yet, previous models relied on an artificial neural network, the basic computational unit of which mimics a real neuron with limited biological realism (Maass, 1997) and communicates using synchronous and continuous signals instead of spikes.

To advance the biological realism of computational models of PC and move toward a more biologically plausible model of perception, we developed a spiking neural network for predictive coding (SNN-PC) by introducing two novel features: (1) a spiking neuron model (Maass, 1997; Gerstner, 2002) that describes the behavior of neurons with more biological details than firing-rate based artificial neurons, such as using binary, asynchronous spikes for synaptic communication and replacing a simple non-linear activation function with synaptic and membrane potential dynamics; and (2) a feedforward gist (FFG) pathway that is added to a PC hierarchy, and mimics the gist of a scene or image (Oliva and Torralba, 2006), inspired by how the visual cortical system may rapidly recognize objects using a fast feedforward visual pathway (Thorpe et al., 1996; Serre et al., 2007; VanRullen, 2007). While our primary goal is to build a spiking neural network that learns a generative model of input image patterns (i.e., to perform image reconstructions) via predictive coding with biologically plausible mechanisms, we also investigate whether such a generative model can be used for a discriminative task (i.e., classification) despite having no explicit objective to optimize it. We hypothesize that having a coarse-level prior about incoming stimuli via the FFG pathway would help with forming classifiable latent representations. In the following sections, we describe non-trivial problems in implementing a spiking version of PC networks, such as encoding signed signals with binary spikes and finding error gradients for experience-dependent learning, and offer our biologically plausible solutions to make PC compatible with spikes. We show that, by putting together all the pieces, SNN-PC can learn hierarchical representations of MNIST hand-written digit images and infer unseen samples from spike signals of sensory inputs in an unsupervised manner.

## 2 Materials and methods

The following section is organized into four subsections, which address challenges of implementing a PC neural network with spiking neurons and propose biologically plausible mechanisms to facilitate perceptual inference and learning: (1) introduction of a spiking neuron model; (2) description of synaptic communication between spiking neurons for reliable signal transmission and Hebbian learning; (3) separation of error-computing neurons into two groups to encode signed signals with dynamic binary spikes; and (4) introduction of the FFG pathway to establish informed initial conditions for prediction-generating neurons as opposed to random initialization.

### 2.1 Single neuron model

The behavior of single neurons in SNN-PC was defined by the adaptive exponential integrate-and-fire model (Brette and Gerstner, 2005):


(1) $$\begin{array}{l} C_{m}\frac{dV}{dt}=-g_{L}*\left(V\left(t\right)-E_{L}\right)+g_{L}\Delta T\exp\frac{V\left(t\right)-V_{\theta }}{\Delta T}+I\left(t\right)-a\left(t\right) \end{array}$$

(2) $$\begin{array}{l} \tau_{a}\frac{da}{dt}=c\left(V\left(t\right)-E_{L}\right)-a\left(t\right) \end{array}$$

where *C*_*m*_ is the membrane capacitance, *V*(*t*) the membrane potential, *g*_*L*_ the leak conductance, *E*_*L*_ the leak reversal potential, Δ*T* the slope factor, *V*_θ_ the action potential threshold, *a*(*t*) the adaptation variable, *I*(*t*) the incoming synaptic current, τ_*a*_ the adaptation time constant, and *c* the adaptation coupling parameter. The parameter values are taken from Brette and Gerstner (2005) and listed in Table 1.

**Table 1:** Parameters for the adaptive exponential integrate-and-fire model.

**Parameter**	**Value**	**Unit**
*C*_*m*_	281	pF
*g*_*L*_	30	nS
*E*_*L*_	−70.6	mV
*V*_θ_	−50.4	mV
Δ*T*	2	mV
*t*_*ref*_	2	ms
*c*	4	nS
*b*	0.0805	nA
τ_*a*_	144	ms
τ_*rise*_	5	ms
τ_*decay*_	50	ms

The membrane potential dynamics (Equation 1) is described by a linear leak, a voltage-dependent exponential activation, which instantiates the fast activation of sodium channels (Badel et al., 2008), the incoming synaptic current, *I*(*t*), and an abstract adaptation variable, *a*, which couples the membrane potential dynamics with voltage-dependent subthreshold and spike-triggered adaptation (Equation 2) (Gerstner et al., 2014).

At each time point of simulation, *t*, a neuron sums up all incoming current, *I*(*t*), at its postsynaptic terminals to update its membrane potential, *V*(*t*). Upon reaching the threshold, *V*_θ_, a spike is generated [i.e., *s*(*t*) = 1]:


(3) $$\begin{array}{l} s\left(t\right)=\{\begin{array}{cc} 1, & \text{ if }V\left(t\right)>V_{\theta }\\ 0, & \text{otherwise} \end{array} \end{array}$$

A spike is followed by an instantaneous reset of the membrane potential, *V*, to the resting potential, (*V*_*r*_ = −70.6 mV), and an increase of adaptation variable (*a*) by an amount (*b*) to model membrane potential repolarization and spike-triggered current adaptation, respectively:


(4) $$\begin{array}{l} s\left(t\right)=1\;\to \;V\left(t\right)=V_{r}\;\&\;a\left(t\right)=a\left(t\right)+b \end{array}$$

### 2.2 Synaptic communication between spiking neurons

The behavior of the single neuron model described in the previous section (Equations 1–4) is a function that takes incoming current, *I*(*t*), as input and a spike train, *s*(*t*), as output. For synaptic communication between spiking neurons, there must be a way to convert the output of a source neuron to input of a target neuron. The current entering a postsynaptic cell *j* (postsynaptic current; PSC) through *N* synapses from presynaptic cells *i* can be formulated as a continuous variable, *I*_*j*_(*t*), by applying an exponential low-pass filter to each incoming binary spike train to compute a spike trace, *X*_*i*_(*t*), and weighting each spike trace with the corresponding synaptic strength, *W*_*i,j*_, and summing the weighted spike traces over *N* synapses (Equation 5):


(5) $$\begin{array}{l} I_{j}\left(t\right)=\overset{N}{\underset{i}{\sum }}W_{i,j}X_{i}\left(t\right) \end{array}$$

The spike trace, *X*_*i*_(*t*), is similar to the trace variable commonly used in spike timing dependent plasticity (STDP). With a proper choice of time constants, it approximates a generic excitatory postsynaptic current (EPSC) that reflects both the fast component driven by AMPA receptors (τ_*rise*_ = 5 ms) and the slow component mediated by NMDA receptors (τ_*decay*_ = 50 ms) (Forsythe and Westbrook, 1988; McBain and Dingledine, 1993) as follows:


(6) $$\begin{array}{l} \frac{dX}{dt}=\frac{Y\left(t\right)}{\tau_{rise}}-\frac{X\left(t\right)}{\tau_{decay}} \end{array}$$

In particular, the NMDAR-mediated component of the EPSC (“NMDAR current”) can be linked to the intracellular calcium concentration at the postsynaptic site, a high value of which leads to long term potentiation (LTP) (Barria and Malinow, 2005; Granger and Nicoll, 2014). Using the NMDAR currents of pre- and postsynaptic neurons, synaptic weights are adjusted by Hebbian learning. The modification of weights is described in a subsequent section.

The first term in Equation (6) governs the rising slope of the EPSC corresponding to the influx of cations into the postsynaptic neuron, whereas the second term describes its decay. The instantaneous reset of the variable, *Y*(*t*), initializes glutamate release into the synaptic cleft, where it binds to AMPA and NMDA receptors to open up ion channels in the postsynaptic membrane (Equation 7):


(7) $$\begin{array}{l} s\left(t\right)=1\;\to \;Y\left(t\right)=1 \end{array}$$

The glutamate concentration in the synaptic cleft decays exponentially back to the resting state [i.e., *Y*(*t*) → 0] (Equation 8):


(8) $$\begin{array}{l} \frac{dY}{dt}=-\frac{Y\left(t\right)}{\tau_{decay}} \end{array}$$

**Figure 1:**
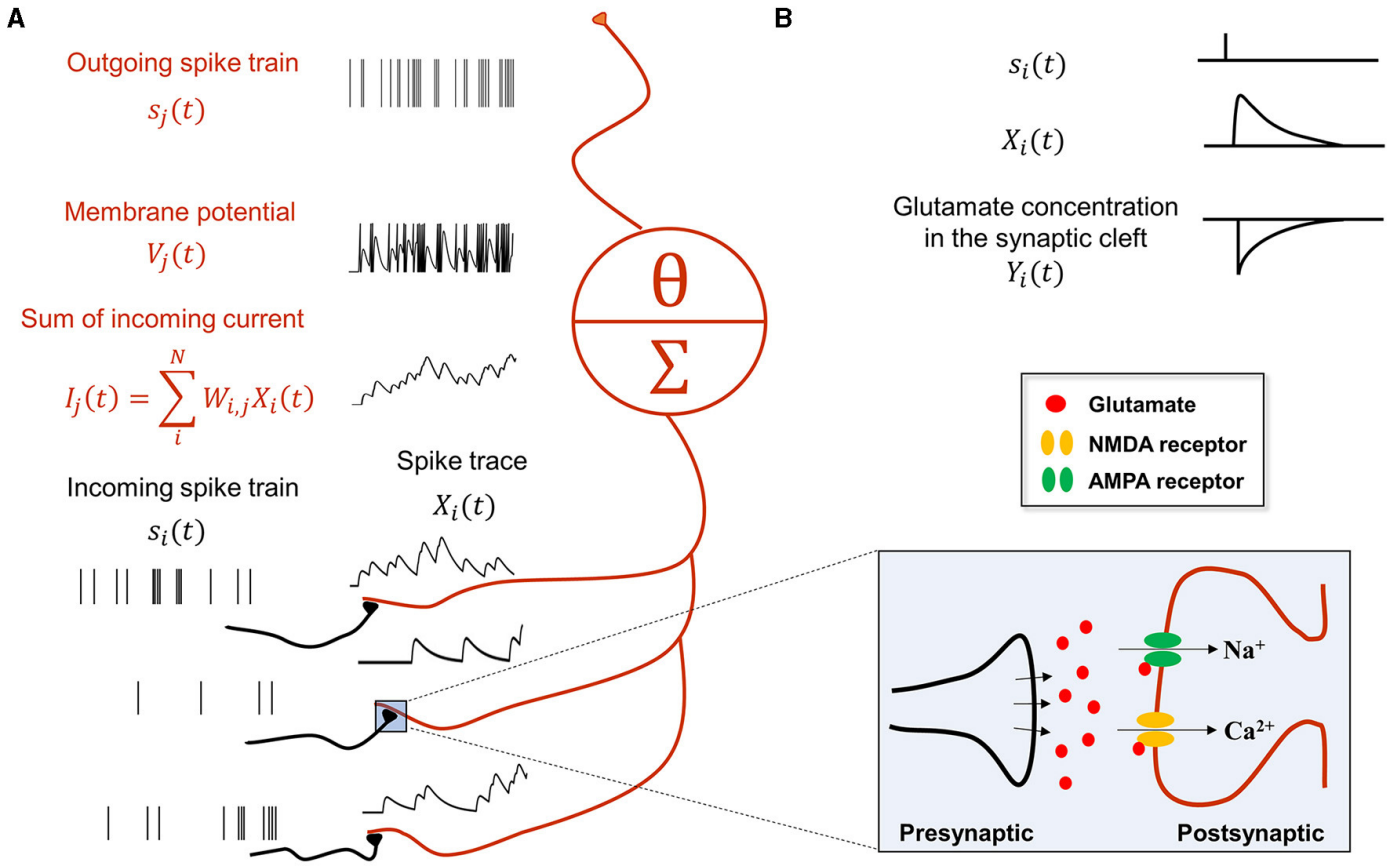
Synaptic transmission in a spiking neural network for predictive coding. **(A)** Schematic showing how spiking neurons in SNN-PC communicate with each other using spikes. Spikes from neurons *i* (black), *s*_*i*_(*t*), activating synapses that impinge on dendrites of neuron *j* (red), are converted to spike traces, *X*_*i*_(*t*). The sum of all spike traces, weighted by synaptic strength, *W*_*i,j*_, make up the postsynaptic current, *I*_*j*_(*t*). In this particular scheme, we assume that all weights are 1 for simplicity. The postsynaptic membrane potential, *V*_*j*_(*t*), changes according to the incoming current and the cell emits spikes, *s*_*j*_(*t*), whenever it reaches threshold, *V*_θ_. **(B)** A presynaptic spike (*s*_*i*_(*t*) = 1) triggers glutamate release into the synaptic cleft [*Y*_*i*_(*t*) = 1]. When glutamate binds to the postsynaptic AMPA and NMDA receptors, the inward current of cations (Na+ and Ca2+) depolarizes the postsynaptic cell (K+ efflux not shown here for brevity). Concentrations of glutamate in the synaptic cleft and cations (Na+ and Ca2+) in the postsynaptic terminal decrease exponentially [*Y*_*i*_(*t*) → 0 and *X*_*i*_(*t*) → 0, respectively] with time constants, τ_*rise*_ and τ_*decay*_, respectively.

In summary, the synaptic communication consists of three major steps (Equations 9–11), which can be seen as a serial adaptation of the spike emission and reception filters in the spike response model (Gerstner et al., 2014). For example, consider the following case where presynaptic neurons (indexed by *i*) project to a postsynaptic neuron *j* (Figure 1):

First, a spike train from neuron *i*, *s*_*i*_(*t*), is converted to an AMPA- and NMDA-receptor mediated postsynaptic current received by neuron *j*, *I*_*j*_(*t*):


(9) $$\begin{array}{l} h_{1}:s_{i}\left(t\right)\mapsto I_{j}\left(t\right) \end{array}$$

Second, the current arriving at the postsynaptic receptor site of neuron *j*, *I*_*j*_(*t*), influences the membrane potential of neuron *j*, *V*_*j*_(*t*):


(10) $$\begin{array}{l} h_{2}:I_{j}\left(t\right)\mapsto V_{j}\left(t\right) \end{array}$$

Third, the membrane potential, *V*_*j*_(*t*), generates a spike, *s*_*j*_(*t*), when it crosses the threshold, *V*_θ_:


(11) $$\begin{array}{l} h_{3}:V_{j}\left(t\right)\mapsto s_{j}\left(t\right) \end{array}$$

### 2.3 Implementation of predictive coding

**Figure 2:**
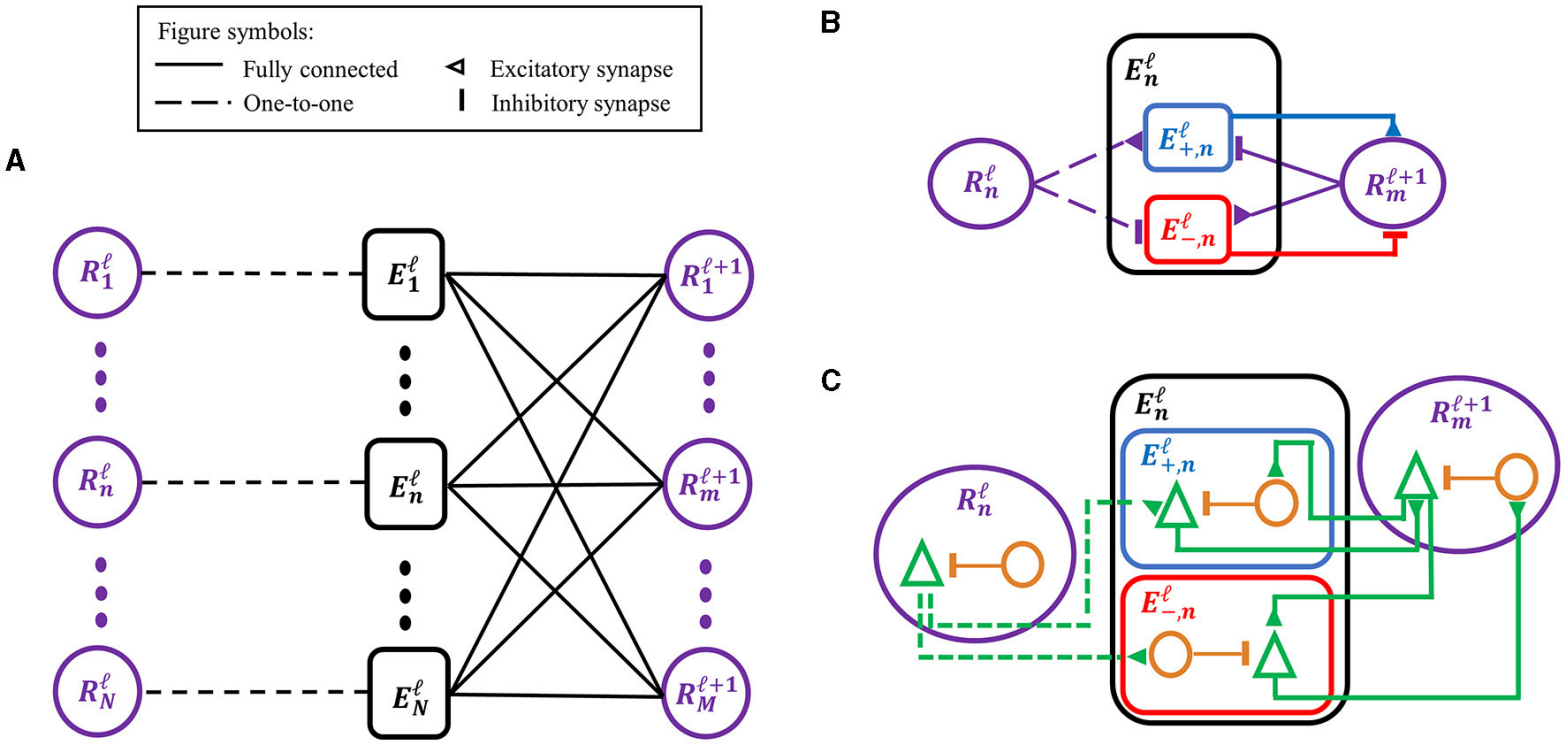
Adaptations of the classic predictive coding (PC) architecture to different levels of biological detail. **(A)** The classic PC architecture with representation (prediction) and error neurons as described by Rao and Ballard (1999). **(B)** To encode signed error signals with binary spikes, an error unit in A, $E_{n}^{\ell }$, is separated into two units, $E_{+,n}^{\ell }$ and $E_{-,n}^{\ell }$, which compute a positive and negative error, respectively, via the opposite arrangement of excitatory-inhibitory synapses. The superscript (ℓ) denotes the cortical processing area, whereas the subscript (n or m) denotes an index for unit in an area. **(C)** A biological interpretation of a computational unit in SNN-PC, which consists of a pyramidal cell (green triangle) and an interneuron (orange circle). An excitatory synapse exists between pyramidal cells of two units, whereas a polysynaptic inhibitory connection is formed by a pyramidal cell in a computational unit to an interneuron in another unit, which inhibits the pyramidal cell within the same unit.

SNN-PC employed the same hierarchical structure as its two predecessors (Figure 2A) (Rao and Ballard, 1999; Dora et al., 2021). Each area (denoted by superscript ℓ) consists of two types of computational units (denoted by subscript *i*): (1) a representation unit, $R_{i}^{\ell }$, which infers the causes (i.e., generates latent representations) of incoming sensory inputs in the area below ($R_{i}^{\ell -1}$) and makes predictions about the neural activity in the area below; and (2) an error unit, $E_{i}^{\ell }$, which compares the prediction from the area above with inputs from the area below and propagates the difference (i.e., prediction error) to the representation units in the area above ($R_{i}^{\ell +1}$) to update the inferred causes and refine the prediction.

#### 2.3.1 Error unit

An error unit computes prediction errors by taking the difference between the sensory input in the lowest area, or its latent representation in the case of higher areas, and the corresponding prediction from the area above. Depending on the relative strengths of the two signals, this difference can be positive (i.e., input > prediction) or negative (i.e., prediction < input). The signed nature of the prediction error poses no obstacle to PC models with artificial neurons, which can encode positive and negative signals. However, given the non-negative nature of spike signals, SNN-PC has to adopt a different solution that can encode both types of prediction error. As observed in the dopaminergic system, a neuron may encode both types of prediction error by expressing the magnitude of error in relation to its baseline firing rate: positive errors are encoded with activity above its baseline firing rate and negative errors with activity below (Schultz et al., 1997). However, such a neuron would require a very high spontaneous firing rate to encode the full range of negative responses, which contrasts with experimental evidence that suggests low baseline firing rates of layer 2/3 principal neurons (De Kock et al., 2007; Perrenoud et al., 2016). Moreover, in a system where single neurons encode bi-directional errors, a postsynaptic neuron that receives error signals must have a mechanism to subtract out the baseline firing rate of a presynaptic neuron. Given the discrete and non-linear dynamics of spiking neurons, which renders accurate approximation of synaptic transmission non-trivial, our attempts to implement bi-directional error coding with spiking neurons led to inaccurate propagation of prediction errors. Therefore, we separated the error unit into two subtypes, one coding positive and the other coding negative error (Figure 2B). The two units are complementary in propagating prediction errors to representation units in the next higher area. A positive error unit (Equation 12) integrates bottom-up excitatory inputs from representation units within the same area, $X_{R}^{\ell }\left(t\right)$, and top-down inhibitory inputs from representation units of the area above, ${\left(W^{\ell ,\ell +1}\right)}^{T}X_{R}^{\ell +1}\left(t\right)$, whereas a negative error unit (Equation 13) has the opposite arrangement of excitatory-inhibitory synapses:


(12) $$\begin{array}{l} I_{E+}^{\ell }\left(t\right)=X_{R}^{\ell }\left(t\right)-{\left(W^{\ell ,\ell +1}\right)}^{T}X_{R}^{\ell +1}\left(t\right) \end{array}$$

(13) $$\begin{array}{l} I_{E-}^{\ell }\left(t\right)={\left(W^{\ell ,\ell +1}\right)}^{T}X_{R}^{\ell +1}\left(t\right)-X_{R}^{\ell }\left(t\right) \end{array}$$

Note that top-down predictions to both positive and negative units are the same (${\left(W^{\ell ,\ell +1}\right)}^{T}X_{R}^{\ell +1}\left(t\right)$). Representation units and the two types of error unit within an area contain the same number of cells and connect to each other in a one-to-one fashion (i.e., $W_{i,j}^{\ell ,\ell }=1$ where *i* = *j* and 0 elsewhere), so that error units receive the same bottom-up input, or its latent representation in the case of higher areas, and compare it to the top-down prediction.

#### 2.3.2 Representation unit

Representation units infer the causes of sensory input via local interactions with error units in the area immediately below as well as those in the same area (Figure 2A). The interactions between two immediately adjacent areas are considered local, because they do not involve areas further down or up in the hierarchy (at least not directly) as would be commonly used in standard deep learning algorithms such as BP, which require global interactions from the top area to the lowest area. The inference process can be regarded as an iterative process of updating internal representations of sensory stimuli (or of neural activity of representation units, $I_{R}^{\ell }$), and is mathematically formalized as performing a gradient descent on the cost function of prediction error minimization with respect to the internal representation (Rao and Ballard, 1999). In SNN-PC, this first comes down to a sum of incoming synaptic current to each representation neuron:


(14) $$\begin{array}{l} I_{R}^{\ell }\left(t\right)=\beta_{+}^{\ell -1}-\beta_{-}^{\ell -1}-\beta_{+}^{\ell }+\beta_{-}^{\ell }\left(0<\ell <L\right) \end{array}$$

The first two terms in Equation (14) represent the bottom-up positive and negative prediction error ($\beta_{+}^{\ell -1}$ and $\beta_{-}^{\ell -1}$), computed as weighted sums of postsynaptic currents arising from the connections between the two error units and representation units, respectively (Equations 15, 16):


(15) $$\begin{array}{l} \beta_{+}^{\ell -1}=W^{\ell -1,\ell }X_{E+}^{\ell -1}\left(t\right) \end{array}$$

(16) $$\begin{array}{l} \beta_{-}^{\ell -1}=W^{\ell -1,\ell }X_{E-}^{\ell -1}\left(t\right) \end{array}$$

The last two elements of Equation (14) are top-down positive and negative errors ($\beta_{+}^{\ell }$ and $\beta_{-}^{\ell }$), respectively, which are connected to representation units in a one-to-one fashion (Equations 17, 18):


(17) $$\begin{array}{l} \beta_{+}^{\ell }=X_{E_{+}}^{\ell }\left(t\right) \end{array}$$

(18) $$\begin{array}{l} \beta_{-}^{\ell }=X_{E_{-}}^{\ell }\left(t\right) \end{array}$$

**Figure 3:**
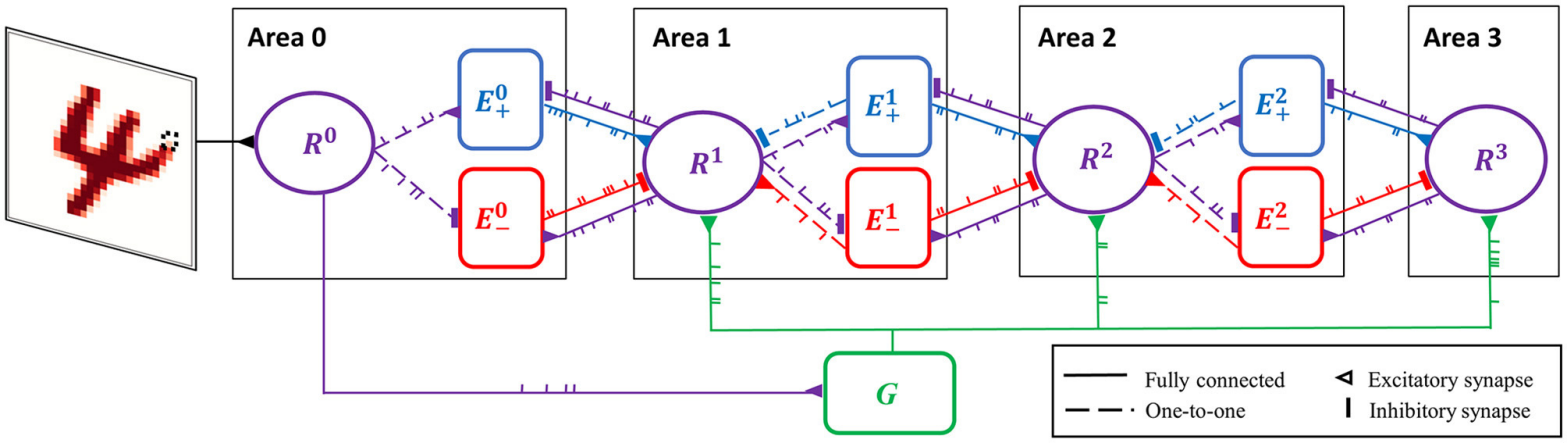
Spiking neural network for predictive coding (SNN-PC). SNN-PC modeling the first stages of the visual cortical processing hierarchy. The three areas roughly correspond to V1, V2, and V4, respectively (or LGN, V1, and V2). Each non-input area (ℓ > 0) consists of a representation unit (purple circle; *R*^ℓ^) and two units (blue and red squares; $E_{+}^{\ell }$ and $E_{-}^{\ell }$) that encode positive and negative prediction error, respectively. The representation unit in Area 0 acts as an input unit (*R*^0^). Each pixel (e.g., the dotted box in the image of digit 4) is encoded by a single spiking neuron. Note that each unit consists of multiple spiking neurons. The feedforward gist pathway (*R*^0^ → *G* → *R*^ℓ^) approximates the feedforward sweep of neuronal activity across the visual processing hierarchy. The solid lines between units indicate that they are fully connected, whereas the dotted lines indicate one-to-one connections. A triangle represents an excitatory synapse, whereas a thick vertical short ending represents an inhibitory synapse.

In the case of the highest area (e.g., Area 3 in Figure 3), these two terms are absent as it lacks top-down connections.

Sensory inputs are fed into the network via representation units in the lowest area (Area 0), each of which receives a constant current linearly proportional to the intensity of a pixel of the input image (Figure 3). The underlying assumption is that such a transduction is roughly comparable to a retinal image (with a resolution of a pixel). Given an MNIST digit sample (28 × 28 pixel image) as visual input, the number of units in Area 0 is 784.

### 2.4 Feedforward gist pathway

Visual cortical processing can be parsed into two distinct processes (Lamme and Roelfsema, 2000): (1) the fast feedforward sweep of neuronal activity across the visual processing hierarchy roughly within 150 ms of stimulus onset in primates, which is thought to generate coarse high-level representation of a visual scene and facilitates gist perception and rapid object recognition (Rousselet et al., 2005; Serre et al., 2007; VanRullen, 2007; Liu et al., 2009; Cauchoix et al., 2016); and (2) slow recurrent processing that iteratively refines the representation (a process henceforth referred to as inference). SNN-PC implements the former process with a FFG pathway and the latter process with the PC hierarchy.

**Figure 4:**
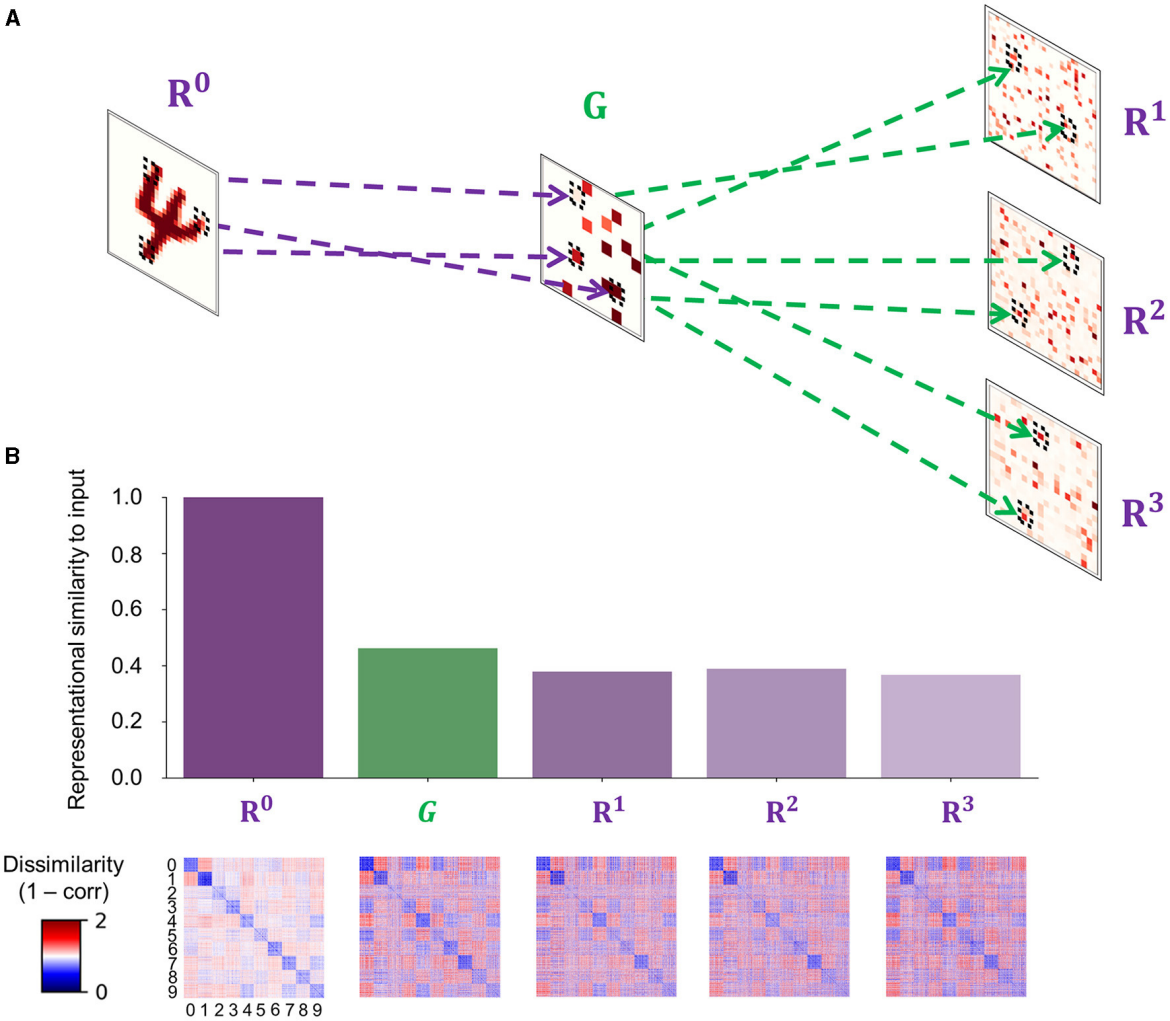
The fast feedforward gist pathway. **(A)** A fast feedforward sweep of neural activity from input (*R*^0^) to representation units (*R*^ℓ^ where ℓ ∈ {1, 2, 3}) via gist units (*G*) is instantiated by sparse random connections to generate a spatially reduced, abstract representation of an input image, which provides an informative baseline for the upcoming iterations of recurrent processing. Purple dashed lines show example projections from input to gist units, whereas green dashed lines show example projections from gist to representation units in areas 1–3 of the visual processing hierarchy. Each tilted square represents an example neural activity pattern activated by an input image in each area. The colored elements in each area correspond to mean incoming synaptic currents. **(B)** A comparison between internal representations formed in areas involved in the feedforward gist pathway (*R*^0^, *G*, and *R*^ℓ^ where ℓ ∈ {1, 2, 3}). Note that representation units only receive synaptic inputs from gist units and exclude other synaptic currents from the recurrent dynamics of the PC hierarchy ($R^{\ell }=W_{G,R^{\ell }}\;X_{G}$). The bar plot displays a measure of similarity between input images and internal representations. Strong correlations indicate that the representational geometry of input images is retained across the FFG pathway (*R*^0^ → *G*) and that representation units in each cortical area (*G* → *R*^ℓ^ where ℓ ∈ {1, 2, 3}) receive informative stimulus-based priors about the upcoming inference. Each element in a representational dissimilarity matrix (RDM) below each bar represents a measure of dissimilarity between 128 different samples per digit (0–9).

The FFG pathway approximates the feedforward sweep across the visual hierarchy via sparse random projections from input to gist units (Figure 4):


(19) $$\begin{array}{l} I_{G}\left(t\right)=W_{I,G}X_{G}\left(t\right) \end{array}$$

The weights between input and gist units (*W*_*I,G*_ in Equation 19) were randomly sampled from a Gaussian distribution, the mean and standard deviation of which were defined as a ratio between the number of pre- and postsynaptic units. To induce sparsity, the connection probability between input and gist units was set to a low value (*P*_*c*_ = 0.05; Figure 4A).

To reflect the increasing receptive field size and complexity of tuning properties when ascending the visual processing hierarchy, the number of gist units (16) is set to be smaller than input units (784). The resulting neuronal activity patterns in gist units therefore correspond to a coarse-grained representation of incoming sensory input. As all input images are processed by the same set of non-plastic, sparse random weights, images that share more features (e.g., two samples belonging to the category of digit “1”) have a higher chance to generate similar neural activity patterns in gist units than those that share less (e.g., a sample belonging to digit “0” and another belonging to “1”); in other words, by statistically sampling the same area in the visual field given different images, the latent representations of input images in gist units retain the representational geometry of input images (Figure 4B).

Gist units then project to representation units in each area of the PC hierarchy to modulate their activity. The synaptic input coming from gist units can be implemented into the inference step (Equation 14) by adding an extra term ($W_{G,R^{\ell }}X_{G}$):


(20) $$\begin{array}{l} I_{R}^{\ell }\left(t\right)=\beta_{+}^{\ell -1}-\beta_{-}^{\ell -1}-\beta_{+}^{\ell }+\\ \beta_{-}^{\ell }+W_{G,R^{\ell }}X_{G}\left(t\right)\left(0<\ell <L\right) \end{array}$$

The FFG pathway runs in parallel with the PC hierarchy and is active as long as the stimulus lasts to provide a high-level, coarse representation of the incoming sensory input to representation units in each PC area ($W_{G,R^{\ell }}X_{G}$; Equation 20) as a baseline activity.

In summary, the FFG pathway serves the role of initializing the neuronal activity of representation units in each area with a coarse representation of the incoming sensory input (e.g., the gist of a scene or object). Instead of starting the iterative process of prediction error minimization from zero or arbitrary activity in representation units, the gist-like latent representation of incoming sensory inputs operates in a biologically plausible manner.

### 2.5 Rate-based Hebbian learning

**Figure 5:**
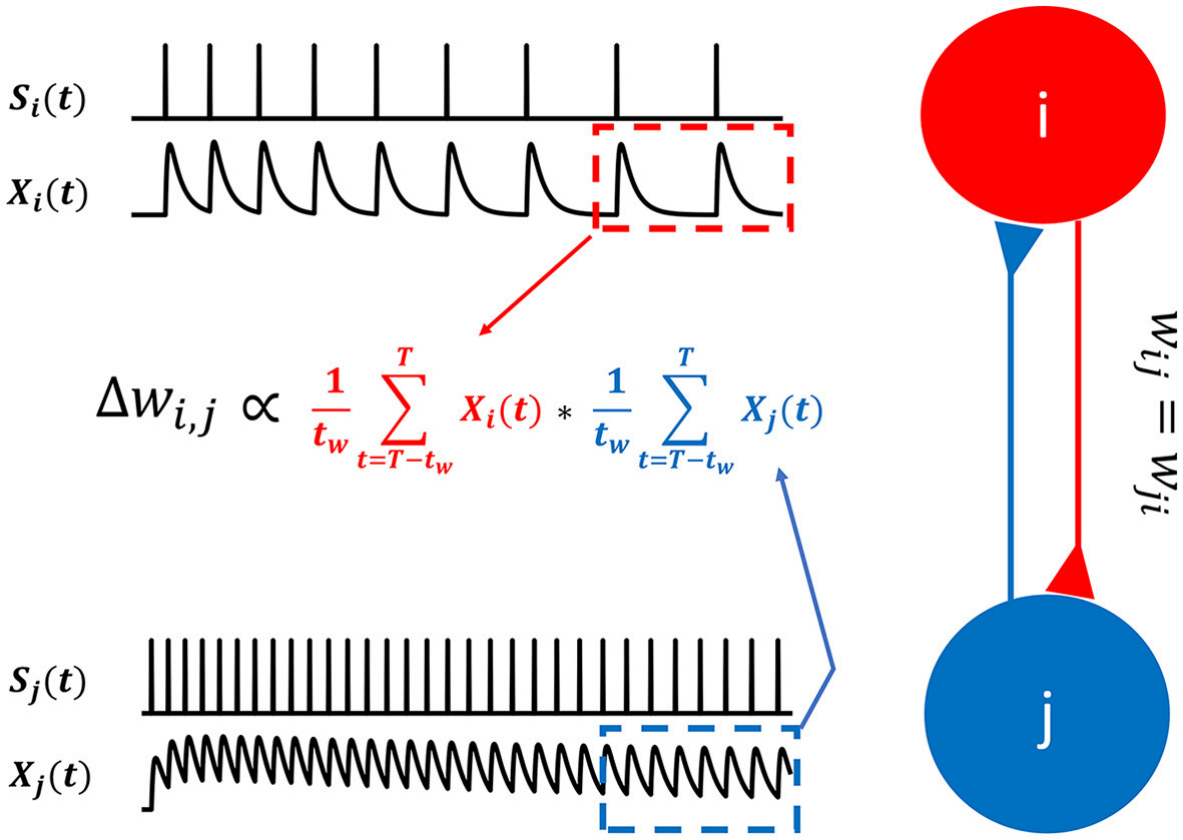
Hebbian learning for spiking neurons. In SNN-PC, synaptic plasticity is mediated by Hebbian learning. Instead of firing rates used in artificial neural networks, SNN-PC approximates NMDA receptor-mediated postsynaptic calcium dynamics, *X*_*i*_(*t*) and *X*_*j*_(*t*), in each cell, based on incoming spike trains, *s*_*i*_(*t*) and *s*_*j*_(*t*), and leverages them to compute a biologically plausible learning gradient, Δ*W*_*i,j*_. The red and blue dotted box indicate the time window, the last *t*_*w*_ ms (from T - *t*_*w*_ to T, where T is the total duration of stimulus presentation), during which the approximate postsynaptic calcium transient signals are averaged.

With non-differentiable binary spike signals, *s*(*t*), the error gradient required to correct the internal model cannot be obtained. However, we can use exponentially filtered spike trains, *X*(*t*), to obtain the ingredients required for Hebbian learning (Figure 5).

A weight matrix, *W*^ℓ,ℓ+1^, between error and representation units (Figure 3), is updated via:


(21) $$\begin{array}{l} \Delta W_{+}^{\ell ,\ell +1}=\frac{1}{t_{w}}\overset{T}{\underset{t=T-t_{w}}{\sum }}X_{E+}^{\ell }\left(t\right)\;\times \;\frac{1}{t_{W}}\overset{T}{\underset{t=T-t_{w}}{\sum }}X_{R}^{\ell +1}\left(t\right)\;\left(\ell <L\right) \end{array}$$

(22) $$\begin{array}{l} \Delta W_{-}^{\ell ,\ell +1}=\frac{1}{t_{w}}\overset{T}{\underset{t=T-t_{w}}{\sum }}X_{E-}^{\ell }\left(t\right)\;\times \;\frac{1}{t_{w}}\overset{T}{\underset{t=T-t_{w}}{\sum }}X_{R}^{\ell +1}\left(t\right)\;\left(\ell <L\right) \end{array}$$

The two terms in Equations (21, 22) are mean NMDAR current amplitudes entering the postsynaptic site of error and representation units from the last *t*_*w*_ milliseconds (ms) of stimulus presentation (total duration = T ms): Equation (22) specifies the use of positive error and Equation (22) of negative error. Apart from convergence and stability purposes to accommodate spiking dynamics, taking this mean reflects the calcium dynamics in dendritic spines, which induce NMDA receptor-dependent long term plasticity and depression (LTP and LTD) (Collingridge and Bliss, 1987; Malenka and Nicoll, 1993; Lüscher et al., 2000). The positive error units contact representation units with excitatory synapses to increase the calcium influx into representation units and can induce LTP, whereas the negative error units make inhibitory synaptic contact to decrease calcium influx and induce LTD (Mulkey and Malenka, 1992). Combining the two (Equations 21, 22) results in a weight update that is a linear combination of the Hebbian error gradients obtained between postsynaptic representation units and the two types of presynaptic error units (Equation 23):


(23) $$\begin{array}{l} \Delta W^{\ell ,\ell +1}=\gamma_{w}\;\left(\Delta W_{+}^{\ell ,\ell +1}-\Delta W_{-}^{\ell ,\ell +1}\right)-\alpha_{w}\;g\left(W^{\ell ,\ell +1}\right)\;\left(\ell <L\right) \end{array}$$

The weight change is controlled by the learning rate, γ_*w*_. The last term, $\alpha_{w}g\left(W^{\ell ,\ell +1}\right)$, models the passive decay of weights by imposing a Laplacian prior on the weights (i.e., L1 regularization) (Dora et al., 2021) (Equation 24):


(24) $$\begin{array}{l} g:x\to \{\begin{array}{cc} 1, & \text{ if }x>0\\ 0, & \text{otherwise} \end{array} \end{array}$$

The resulting unsupervised learning algorithm can be regarded as a biologically plausible form of Hebbian learning. It uses the AMPA- and NMDA-receptor mediated postsynaptic currents between neurons located in adjacent cortical areas (i.e., area ℓ and ℓ + 1), thereby requiring only locally available information. This is in contrast to backpropagation, which often requires explicit labels (supervised learning) and an end-to-end propagation of errors, from individual output units up to input units. Note that only inter-areal weights (*W*^ℓ,ℓ+1^) are subject to synaptic plasticity. The intra-areal weights (*W*^ℓ,ℓ^) are fixed.

### 2.6 Simulation details

#### 2.6.1 Preprocessing of input image

The input images were normalized to unit vectors to limit the variance among pixel intensity distributions across different digits and samples and scaled to a range between 600 and 3,000 pA, within which the input and output synaptic currents approximated a linear relationship.

#### 2.6.2 Network size

Each area consisted of the same number of positive error units, negative error units, and representation units (Area 0 = 784 × 3 = 2,352; Area 1 = 400 × 3 = 1,200; Area 2 = 225 × 3 = 675), except the top area (Area 3) that only contained 64 representation units (Table 2). There were 16 gist units. In total, the number of units in SNN-PC was 4,307. Out of 431,842 total synapses in the network, 418,000 inter-areal synapses were subject to synaptic plasticity.

**Table 2:** Parameters for simulation.

**Parameter**	**Meaning**	**Value**
$n_{R^{0}}$	Number of units in *R*^0^	784
$n_{E_{+}^{0}}$	Number of units in $E_{+}^{0}$	784
$n_{E_{-}^{0}}$	Number of units in $E_{-}^{0}$	784
$n_{R^{1}}$	Number of units in *R*^1^	400
$n_{E_{+}^{1}}$	Number of units in $E_{+}^{1}$	400
$n_{E_{-}^{1}}$	Number of units in $E_{-}^{1}$	400
$n_{R^{2}}$	Number of units in *R*^2^	225
$n_{E_{+}^{2}}$	Number of units in $E_{+}^{2}$	225
$n_{E_{-}^{2}}$	Number of units in $E_{-}^{2}$	225
$n_{R^{3}}$	Number of units in *R*^3^	64
*n*_*G*_	Number of units in *G*	16
*dt*	Simulation time step	1 ms
τ_*w*_	Time window for synaptic plasticity	100 ms
*T*	Total simulation time per sample	350 ms
γ_*w*_	Learning rate for synaptic plasticity	1e-7
α_*w*_	Regularizer for synaptic plasticity	1e-5
$n_{sample}^{train}$	Number of samples in training set	5,120
$n_{sample}^{test}$	Number of samples in test set	1,280
$n_{sample}^{batch}$	Number of samples in a mini-batch	32
$n_{batch}^{epoch}$	Number of mini-batches per training epoch	160
*n*_*epoch*_	Number of training epochs	50

#### 2.6.3 Training and testing

In order to test whether SNN-PC can learn statistical regularities of incoming sensory inputs and build latent representations thereof, we trained SNN-PC with a subset of the MNIST handwritten digit image dataset. The training set ($n_{train}^{sample}=5,120$; Table 2) consisted of many different image samples per image class (digits 0–9; 512 samples / class). For efficient learning, we used mini-batch training ($n_{sample}^{batch}=32$). During a single training epoch, the network goes through all mini-batches ($n_{batch}^{epoch}=160$). After each mini-batch, the synaptic weights are updated. After 50 training epochs (*n*_*epoch*_ = 50), we tested the model to infer image samples it had not been exposed to during the training, taken from a test set ($n_{sample}^{test}=1,280$). For statistical inference, the testing phase was repeated 100 times. Each test set was randomly sampled from 10,000 images that the network had not seen during the training.

For learning, we took the mean over the last 100 ms (300–400 ms) of synaptic current relative to the onset of the stimulus to ensure convergence. Weights were initialized randomly, but strictly positive, by sampling from a half-normal distribution around zero mean and a small standard deviation (0.3). They were updated after every batch with an initial learning rate (γ_*w*_) of 1e-7 and a regularization parameter (α_*w*_) of 1e-5. The learning rate for each pair of areas (e.g., Area 1 and 2) was adjusted by fitting the normalized root mean squared errors of input area (e.g., Area 1) to an exponential growth function.

### 2.7 Representational similarity analysis

A representational similarity analysis (RSA; Kriegeskorte et al. 2008) computes pairwise similarity between population responses for given inputs. The output of this analysis generates a representational dissimilarity matrix (RDM), each block of which represents the dissimilarity (1 − correlation) between responses to different images. To assess the consistency of information propagation through the processing hierarchy, we conducted RSA on the internal representations of input images ($X_{R}^{\ell }$). We used the Spearman rank correlation coefficient as a measure of correlation distance between internal representations (see Kriegeskorte et al. 2008 for more choices of distance measures). A second-order RSA computes a similarity measure between RDMs and represents how similarly two areas of interest respond to a given set of input patterns, thereby revealing consistency in representational geometry (i.e., how well internal representations reflect the relationship between input images).

## 3 Results

### 3.1 Representational learning

As a generative model with an objective function of prediction error minimization, SNN-PC is expected to generate internal representations of input stimuli, which capture their underlying structures (i.e., probability distribution) in a high-dimensional latent space and, therefore, can be used to reconstruct them. To test this representational capacity, we trained it with a small subset of the MNIST handwritten image dataset (*n*_*class*_ = 10 and *n*_*image*_ = 5120; 8.5% of a full set, which has 60,000 images) and evaluated its internal representations of those images. Moreover, while a two-layer structure suffices for image reconstruction in principle, we investigated the impact of hierarchical dynamics on reconstruction performance and other cognitive functions such as image classification by introducing additional processing areas. Note that classification capacity of learned internal representations is only a serendipitous byproduct of our original goal: learning a generative model. We report that additional hierarchical constraints do not impair or improve reconstruction performance.

**Figure 6:**
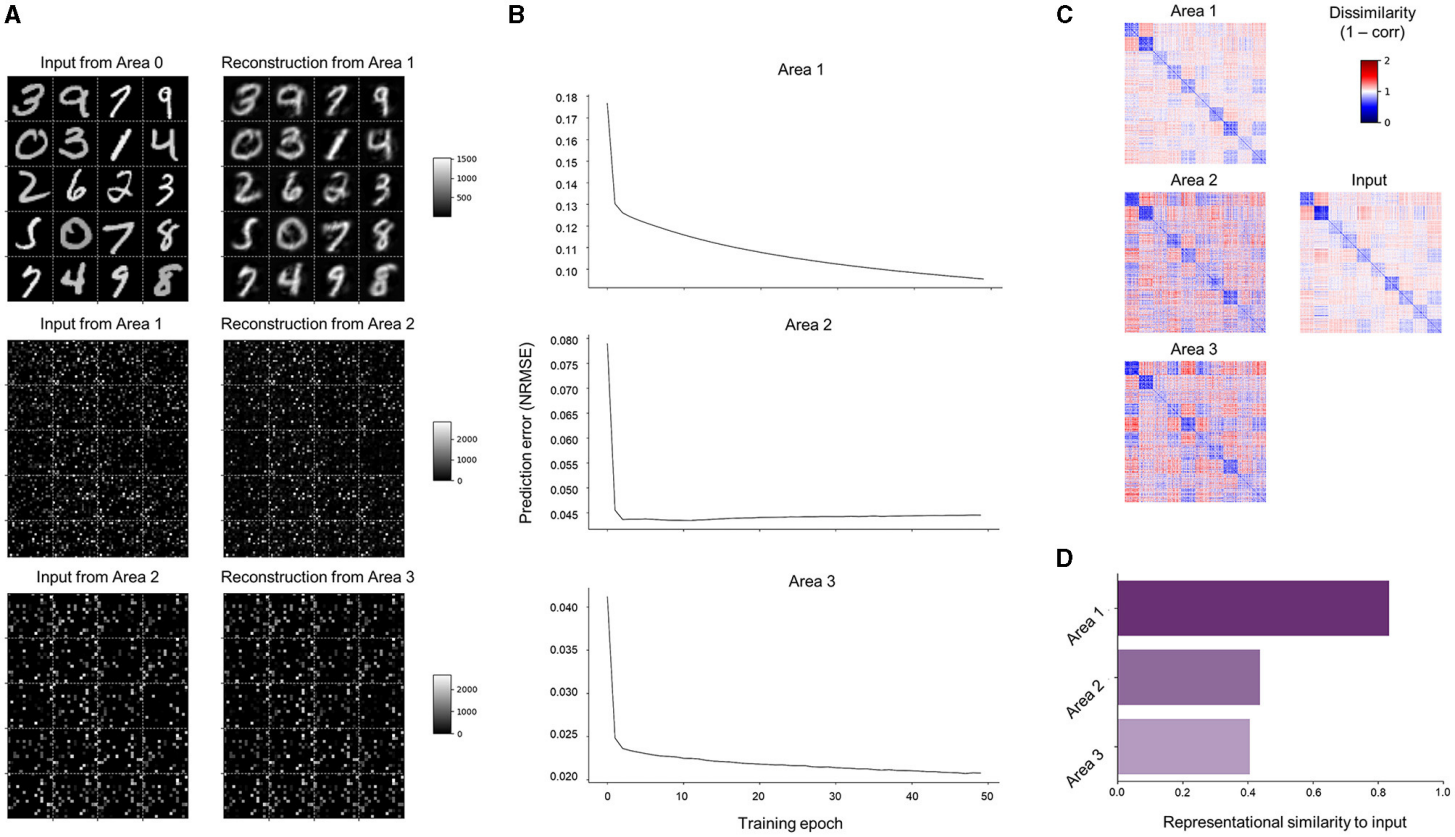
Learning of MNIST handwritten digits. **(A)** Reconstruction of sensory inputs. Input to each area ($X_{R}^{\ell }$; left) is compared to the prediction ($W^{\ell ,\ell +1}\;X_{R}^{\ell +1}$) made from the area immediately above (right). **(B)** Inference. Normalized root mean squared prediction errors (NRMSEs) in each area across training epochs computed by SNN-PC. **(C)** Representational dissimilarity matrices (RDMs) constructed with images in the input area (middle row, right column) and inferred causes of those images in each area from SNN-PC with (left column). Each element in an RDM represents a measure of dissimilarity between them (1 − correlation). **(D)** Second-order representational similarity analysis. The RDM of input neuron activities is compared against the RDMs of inferred causes of those input signals in each area of the SNN-PC model.

Learning performance can first be qualitatively assessed from the error units in the lowest area (Area 0), which receive bottom-up sensory inputs that directly correspond to pixel intensities of input images ($X_{R}^{0}$) and top-down predictions that reconstruct them ($W^{0,1}X_{R}^{1}$). By organizing neural activity patterns that correspond to the predictions (Area 1 → 0) into the input shape (28 × 28 pixels), the images reconstructed by SNN-PC can be visualized. SNN-PC was able to reconstruct digit images well (Figure 6A; top subpanel). While similar inspection in higher areas showed matching patterns between input and prediction (Figure 6A; Area 2 → 1 shown in the middle and Area 3 → 2 shown in the bottom subpanel) as well, predictions in higher areas ($W^{\ell +1,\ell }X_{R}^{\ell }$; Area ℓ + 1 → ℓ where ℓ > 1) bear no immediately recognizable meaning to a human beholder; therefore, they are called latent representations. To show that learning takes place in all areas more evidently, we show the normalized root mean squared errors (NRMSE) as a scale-free measure of the discrepancy between incoming inputs (either sensory or from area 1 to 2 or area 2 to 3) and their predictions. The decreasing NRMSEs in all three areas across training epochs (Figure 6B) indicate that SNN-PC learned to hierarchically minimize prediction errors. Furthermore, a representational similarity analysis (RSA) (Kriegeskorte et al., 2008) on the internal representations of input images ($X_{R}^{\ell }$) revealed that similar representations were formed across the hierarchy. Note that the colored square boxes shown in Figure 6C are representational dissimilarity matrices (RDMs), which illustrate all pairwise dissimilarities (1 − correlation) among the internal representations corresponding to the input images. To quantitatively evaluate how well those internal representations ($X_{R}^{0}$) reflect the relationship between input images (i.e., representational geometry), we conducted a second-order RSA. The results revealed that Area 1 exhibited a high correlation with inputs, whereas Area 2 and 3 displayed weak correlations with inputs (Figure 6D). Furthermore, despite the decreasing prediction errors (Figure 6B; middle and bottom subpanels), reconstructions of input images from Area 2 and 3 [i.e., $W^{0,1}\left(W^{1,2}X_{R}^{2}\right)$ and $W^{0,1}\left(W^{1,2}\left(W^{2,3}X_{R}^{3}\right)\right)$] failed to produce the input images. Hence, for the subsequent analyses in this study, we will focus on the neuronal activities of Area 1 ($X_{R}^{1}$) and refer to them as SNN-PC's internal, latent representation of input images. Possible reasons for the underperformance in higher areas will be examined in the Discussion section. Overall, good reconstruction performance (Figure 6A; top subpanel), proper prediction error minimization (Figure 6B; top subpanel), and strong representational similarity (Figures 6C, D; Area 1) in Area 1 suggest that SNN-PC has successfully extracted statistical regularities from sensory inputs and updated its internal representations to infer their causes more accurately.

### 3.2 Robustness of latent representations

To examine the robustness of the representational capacity of SNN-PC, we tested it on a set of MNIST digit images, which it had not seen during the training (i.e., “Clean” set; *n*_*class*_ = 10 and *n*_*image per class*_ = 128). Subsequently, we challenged the robustness by testing the network on two additional datasets created by modifying the input statistics of the Clean set: (1) the first set (“Noise”) was given additive Gaussian noise, ϵ~N(0,300) (with pA as unit), that spanned across the whole image; and (2) the second set (“Occlude”) was masked by occlusion patches at random locations on images (patch size = 9 × 9 pixel; 10.3% of an image). Note that the network had been trained only on Clean images but tested on all three input variants.

**Figure 7:**
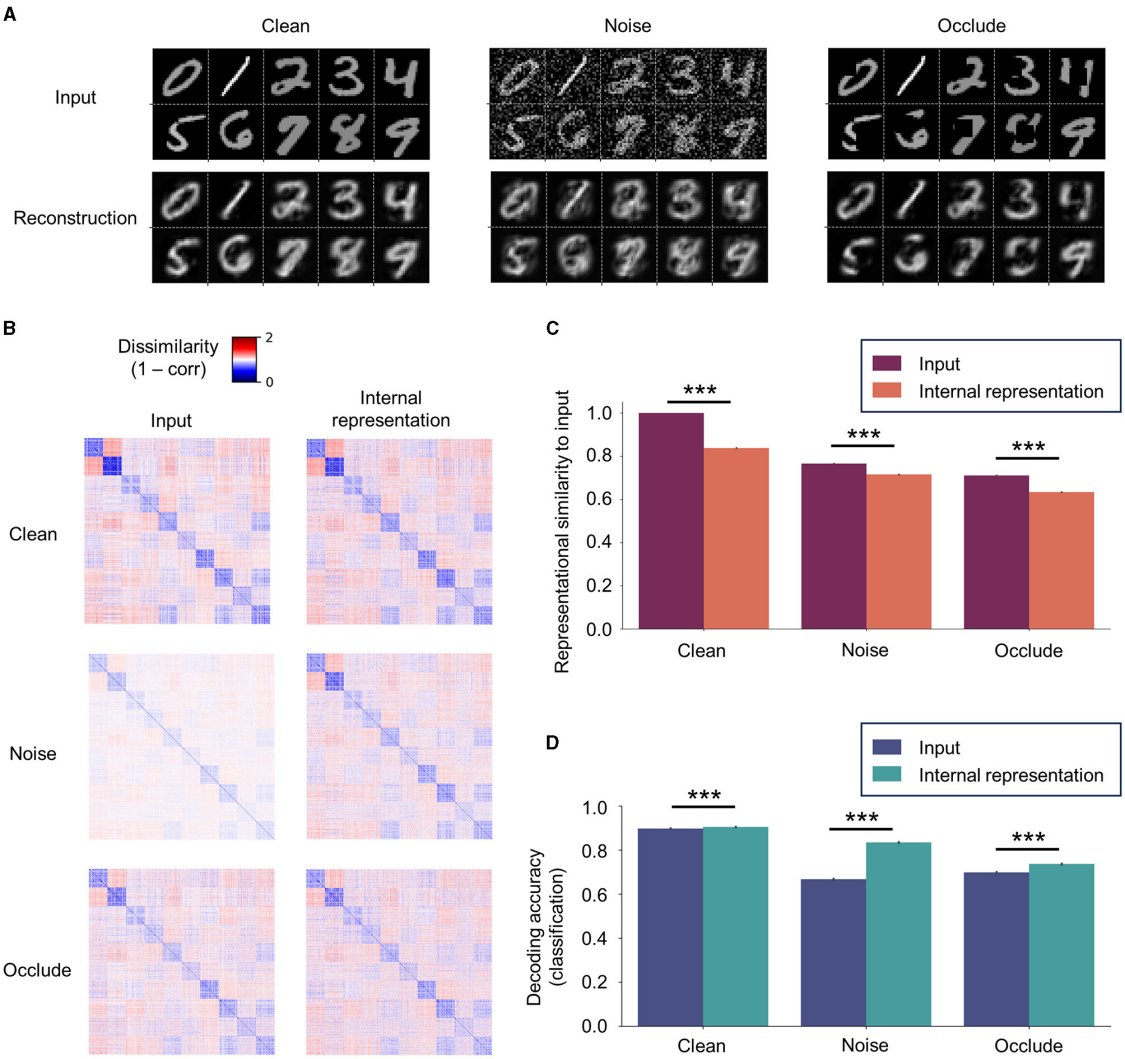
Robustness of internal representations. **(A)** SNN-PC's reconstruction of novel (previously unseen; “Clean”), noisy (“Noise”), and occluded (“Occlude”) images. The network was able to generalize the internal model acquired during training to novel instances and denoise. However, it does not fully restore the occluded part of an image. **(B)** The robustness of internal representations against addition of noise or partial loss of input signals is shown by RDMs among novel input images and corresponding internal representations. **(C)** The internal representations were highly correlated to novel input images (ρ > 0.6 for all bars in **C**). Note that robustness refers to the network's representational capacity to preserve the relationship between input images in the high-dimensional, latent space (i.e., representational geometry) against input perturbations. **(D)** Digit class information can be decoded better from internal representations than input images with or without input perturbations. For statistical comparisons in **(C, D)**, we computed RDMs and classification accuracy on 100 test sets (*n*_*img*_ = 1,280) randomly sampled from the “Clean” test set (*n*_*img*_ = 10,000). Differences between the means were then assessed by Mann-Whitney *U*-test. Statistical significance is indicated by asterisks (***p ≤ 0.001). Error bars indicating 95% confidence intervals. Note that a linear classifier was trained only once on Clean images and internal representations inferred from those images and tested on Clean, Noise, and Occlude dataset images and corresponding internal representations.

We found that SNN-PC was able to generalize the internal model it had acquired during the training onto novel instances as shown by both faithful reconstruction (Figure 7A; Clean) and RDMs that highly correlated with input images (ρ > 0.8, where ρ is Spearman's rank correlation coefficient; Figures 7B, C, Clean). It was also able to denoise (Figure 7A; Noise) and retain representational geometry of input images (ρ > 0.6; Figures 7B, C, Noise). Meanwhile, pixels behind random occlusion patches were not fully restored (Figure 7A; Occlude); thus, the Clean version of the input was not pattern-completed, whereas the occluded input as offered was in fact faithfully reconstructed. However, internal representations generated from partially occluded images still showed strong correlation with input images (ρ > 0.6; Figures 7B, C, Occlude).

The contrast between dissimilarities within and between classes in an RDM reflects the network's capability of encoding sensory inputs into meaningful latent representations; a greater contrast indicates more generalizable representations and often leads to a better classification performance. The reduced contrast in the RDMs of Noise or Occlude input neuron activities compared to those of Clean input neuron activities (Figure 7B; “Clean” vs. Noise and Clean vs. Occlude at input level) reflects the effect of additive Gaussian noise or random patch occlusion on input images. This loss of representational similarity from input perturbations persisted in the RDMs of latent representations (Figure 7C; “Clean” vs. Noise and Clean vs. Occlude at the internal representation level). However, the corresponding internal representations in both input variants were still strongly correlated with original images (ρ > 0.6).

To assess the robustness of the discriminative capacity of SNN-PC against input perturbations, we trained a linear classifier on the internal representations of images from the training set without any perturbations (the same set as in Figure 6; *n*_*class*_ = 10 and *n*_*image per class*_ = 512) and tested it on the images from test sets with (Clean) and without input perturbations (Noise and Occlude). For statistical comparison, Mann-Whitney's *U*-test was used on the classification results of 100 test sets (*n*_*class*_ = 10 and *n*_*image per class*_ = 128) randomly sampled from 10,000 images. Our results revealed that digit class could be decoded better from internal representations than from input images themselves in all three input variants (Figure 7D; *p* < 0.001).

### 3.3 The effect of the feedforward gist pathway

While the faithful input reconstruction, consistent representational geometry, and improved decoding observed with novel (Clean) and corrupted (Noise and Occlude) datasets could indicate meaningful representation learning of input statistics, the effect of the FFG pathway remained elusive thus far. For instance, the network could also have learned to build an internal model entirely based on feedforward gist inputs, thereby rendering the recurrent dynamics of PC redundant, or vice versa. To investigate the contribution of the FFG pathway to perceptual inference and learning, we trained another network on the same training set without the FFG pathway (“PC-only”), which learns the underlying structures of input images purely from the recurrent dynamics of PC. We compared reconstruction, representational geometry, and classification results of the PC-only model against those of the original model (“PC + FFG”), which employs both the PC hierarchy and the FFG pathway, and the FFG pathway model (“FFG-only”), in which input signals were processed only by the FFG pathway.

**Figure 8:**
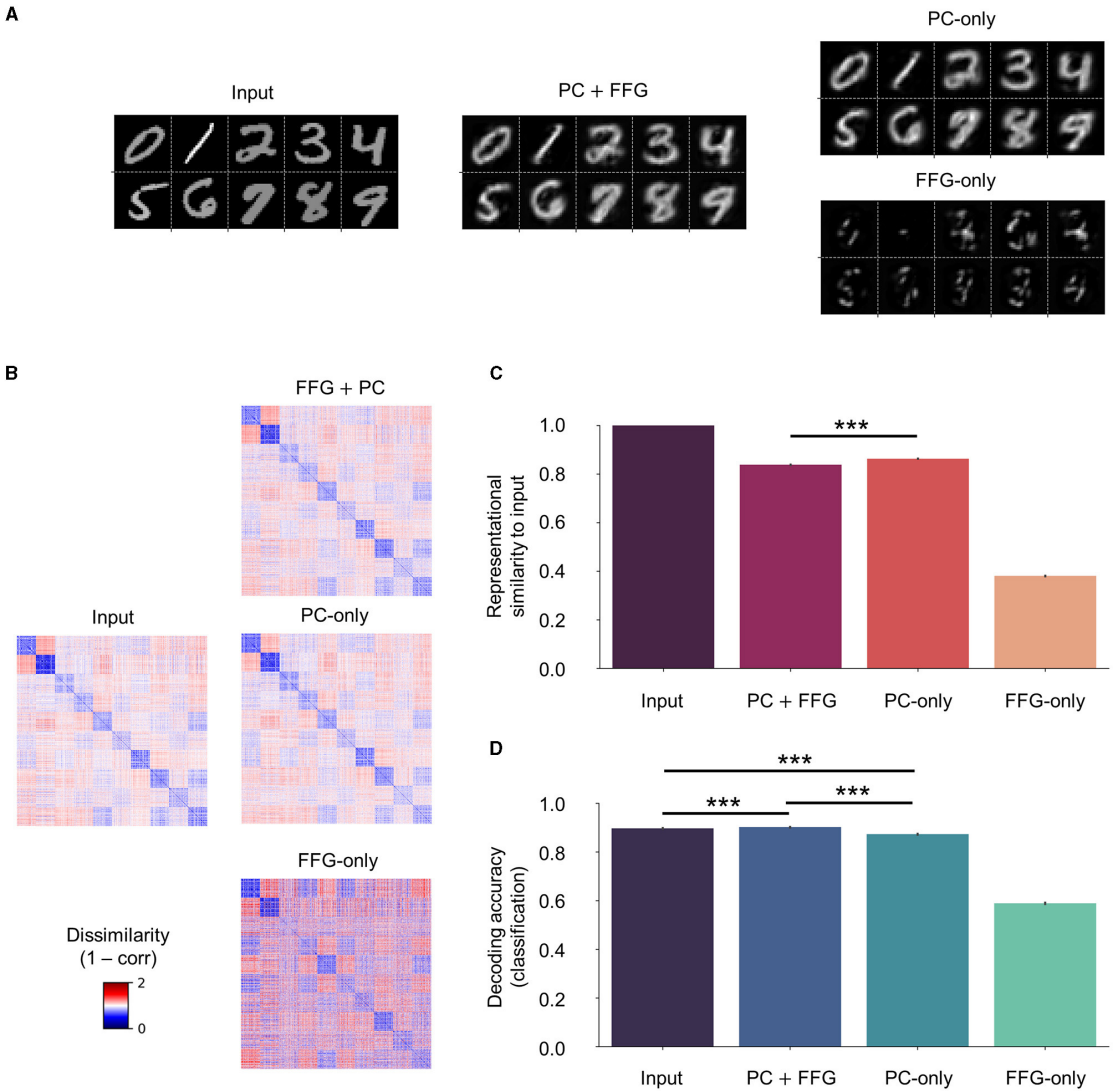
Effect of the feedforward gist pathway on representational learning. Three models were compared against each other on input reconstruction, representational geometry, and classification: (1) a model that processes input stimuli via both the PC hierarchy and the FFG pathway (“PC + FFG”); (2) a model that utilizes only the PC hierarchy (“PC-only”); and (3) the FFG pathway model (“FFG-only”). **(A)** Input reconstruction is a result of the recurrent dynamics of PC (PC + FFG and PC-only). The FFG pathway alone cannot account for the generative capacity (FFG-only). **(B)** PC preserves the metric relationship (1 − correlation) among input images in the representational geometry. **(C)** In the presence of PC, internal representations show a strong correlation with inputs (ρ > 0.8). The FFG pathway alone generates coarse, gist-like representations that only weakly correlate to input images (ρ < 0.4). **(D)** The FFG pathway helps slightly but significantly in inferring internal representations from input images that are more discriminative of digit class than PC-only. For statistical comparisons in **(C, D)**, we computed RDMs and classification accuracy on 100 test sets (*n*_*img*_ = 1,280) randomly sampled from the “Clean” test set (*n*_*img*_ = 10,000). Differences between the means were then assessed by Mann-Whitney *U*-test. Statistical significance is indicated by asterisks (***p ≤ 0.001). Error bars indicating 95% confidence intervals. Note that four separate linear classifiers were trained on input images and internal representations inferred from those images by the three model variants.

Without PC, the FFG pathway could not faithfully reconstruct input images (Figure 8A; FFG-only), formed internal representations that were only weakly correlated to input images (ρ < 0.4; Figures 8B, C; FFG-only), and performed poorly on a digit classification task [decoding accuracy of FFG-only model or DA (FFG-only) ≈0.6; Figure 8D; FFG-only]. These results were consistent with our modeling objectives for the FFG pathway: it was designed to generate only a coarse representation of input images. Note that, to visualize the gist-like internal representations, we reconstructed input images using synaptic weights from a model trained with both FFG and PC (PC + FFG).

On the other hand, PC could reconstruct input images in the absence of FFG just as well as in its presence (Figure 8A; PC-only). In fact, internal representations correlated significantly more with input images without than with FFG inputs to the network [ρ (PC-only) > ρ(PC + FFG) with *p* < 0.001; Figure 8B]. However, a model classified the digit class of images better when it had learned statistical regularities of those images with both PC and FFG pathway than PC alone [DA (PC + FFG) > DA (PC-only) with *p* < 0.001; Figure 8D].

The reconstruction of sensory inputs was achieved with or without the FFG pathway, because internal representations are inferred via the PC hierarchy which is configured to minimize the difference between sensory inputs and a linear transformation of internal representations (i.e., prediction error). However, the difference in representational similarity to input images between the two cases (Figure 8C; PC + FFG vs. PC-only; *p* < 0.001) suggests that they did not converge on the same internal representations, despite both preserving the representational geometry of input images (ρ > 0.8 in both cases). The only difference between the two cases in how they inferred internal representations of input images was the prior: with the FFG pathway, representation units are provided with informative priors about input images through sparse connections (ρ > 0.3; Figure 8C; FFG-only); whereas a purely PC network assumes uniform priors about input images. In sum, our results suggest that the FFG pathway aids representational learning via PC by providing informative priors about sensory inputs for the upcoming inference process, helping to infer internal representations that are more discriminative of class information than the uniform priors of PC alone. Effectively, the FFG can be said to install a prior in the network based on which the PC machinery refines the representation.

## 4 Discussion

While inspired by biological neurons, artificial neural networks make many assumptions for the sake of functional and computational efficiency. For instance, the assumption underlying the use of firing rates as a measure of neural activity is that information is rate coded. However, the brain is also thought to encode information using both the timing of spikes (e.g., phase coding and neural synchrony) (Gray et al., 1989; O'Keefe and Recce, 1993; Singer, 1999; Knoblauch and Palm, 2001; Van Rullen and Thorpe, 2001; Brette, 2012; Ono and Oliver, 2014) and aggregate responses of neuronal ensembles (i.e., population coding) (Georgopoulos et al., 1986; Lee et al., 1988; Pouget et al., 2000; Averbeck et al., 2006). By collapsing the temporal dimension into an arbitrary iteration step, artificial neurons cannot leverage asynchronous, event-based, and sparse information processing for energy efficiency (Pfeiffer and Pfeil, 2018; Tavanaei et al., 2019; Deng et al., 2020). Moreover, the non-local, end-to-end error propagation of BP, often used in ANNs, poses a serious challenge to biological plausibility (Rumelhart et al., 1986; Bengio et al., 2015; Sacramento et al., 2018; Whittington and Bogacz, 2019; Lillicrap et al., 2020; Song et al., 2020). To create a realistic system that performs complex cognitive behaviors on par with a human agent, we need to study and incorporate principles of neural computation and architectures from the biological agent we want to mimic, namely the mammalian brain. A straightforward choice in pursuing such endeavors therefore was to introduce spiking neurons, as they provide a biophysically realistic level of detail to simulate basic computations in the brain.

To this end, we developed a biologically grounded neural network for generative visual modeling (SNN-PC), based on the following four components: (1) a predictive coding model that provides computational algorithms and a neural architecture for generative perceptual inference via recurrent sensory processing; (2) a FFG pathway that accounts for rapid feedforward processing in the visual cortical system; (3) spiking neurons that reflect the time-varying pulsatile behavior of neurons better than rate-based neurons; and (4) Hebbian learning enabled by NMDAR-mediated synaptic plasticity. The model learned to reconstruct and develop latent representations of the MNIST handwritten digit images using only a small subset of the image dataset (8.5% of the full dataset) and an unsupervised learning method that requires only locally available information at each level of the hierarchy (as opposed to end-to-end backpropagation). Furthermore, our implementation of PC is based on biologically grounded mechanisms such as an adaptive spike generation mechanism, synaptic transmission modeling the effect of presynaptic spikes on the postsynaptic membrane potential, and synaptic plasticity based on calcium transients in postsynaptic dendritic spines.

### 4.1 Robustness against noise and occlusion

While previous studies have explored robustness of PC network's generative capacity against noise and partial occlusion, both denoising and pattern completion require structural modifications such as lateral connections and auxiliary connections between non-local areas (Ororbia, 2023) or algorithmic adjustments such as adding a memory vector and unclamping input units of the missing pixels from an input image and allowing it to vary from top-down prediction (Salvatori et al., 2021) or conditional inference on pre-trained labels (Salvatori et al., 2022). Our results exhibit a contrary case, where the missing part is not filled in by the top-down prediction; rather, the SNN-PC infers faithfully from the actual sensory inputs (i.e., with occlusion). This is because, without structural or algorithmic modifications, the local prediction error minimization loop always aligns predictions to inputs. Also, assuming that the reconstruction of sensory inputs from Area 1 of SNN-PC is a direct prediction of the retinal image, the pattern completion should not occur as a subject never actually sees the occluded part. For example, if you walk on a street and find an uncovered manhole, it is not in your interest to fill it in based on priors built upon previous encounters with covered manholes. However, the digit class information was better decoded from internal representations of occluded images in the latent space than from actual images themselves (Figure 7D; Occlude). This implies that SNN-PC was able to capture underlying structures of digit images during training, which were indeed robust against a partial loss of pixels. A recent study (Papale et al., 2023) showed comparable experimental evidence to our results: multi-unit spiking activity of monkey V1 neurons exhibited a significantly weaker response to occluded regions than non-occluded regions; nevertheless, the scene information could be decoded from a cross-decoding experiment (i.e., training on non-occluded images and testing on occluded images).

Additionally, SNN-PC was able to denoise. While structural similarity index measure (SSIM; Wang et al., 2004) values decrease with an increasing level of noise, the network was able to denoise up to a high level of Gaussian noise, and the reconstruction performance did not acutely break down within a range of 0–200 % (figure not shown here). Given the nature of an inference model that builds upon statistical regularities of input signals, SNN-PC simply cannot make predictions about noise, which by definition is unpredictable. Hence, it leveraged on those input signals that could be predicted and showed denoised reconstructions, robust internal representations, and better decodability from internal representations than noisy images themselves.

### 4.2 Novel features of the predictive coding model with spiking neurons

To our knowledge, no predictive coding model has been proposed before that is operating purely with spiking neurons, except for the spiking neural coding network (SpNCN) proposed in Ororbia (2023). While SNN-PC and SpNCN similarly implement synaptic transmission (i.e., low-pass filtering of spike trains) and weight updating (i.e., Hebbian learning), a few key differences arise from the additional biological constraints we placed on our model. For example, spiking neurons in SNN-PC are based on the AdEx model, which offers a biophysically more accurate description of a neuron's behavior than the leaky-and-integrate fire (LIF) model used in SpNCN (Brette and Gerstner, 2005). However, the most noteworthy difference is that error units in SNN-PC are explicitly modeled as spiking neurons and separated into two groups to encode both positive and negative error with binary spikes, as opposed to being an arbitrary unit that signals a signed difference between two exponentially filtered spike trains, as in Ororbia (2023). While such an arbitrary error unit might be biologically implemented in a dendritic compartment model (e.g., Urbanczik and Senn 2014; Mikulasch et al. 2023), our implementation of error neurons follows experimentally observed mismatch between feedforward and feedback signals in visual cortical neurons (e.g., of the somatostatin-positive type) (Keller et al., 2012; Zmarz and Keller, 2016; Attinger et al., 2017). Moreover, having two types of error unit to compute positive and negative error separately also circumvents the need to employ unrealistic negative synaptic weights for inhibitory connections (Figure 2B). A growing body of recent studies suggests that layer 2/3 of cortex indeed contains neurons that express positive or negative errors (Keller et al., 2012; Jordan and Keller, 2020; O'Toole et al., 2023).

Despite having solved the implausible negative weight problem, all units in SNN-PC (when viewed as single neurons) do not adhere to another biological property (i.e., Dale's principle) as they can form both excitatory (in case of the $R^{\ell }\to E_{+}^{\ell -1}$ projection) and inhibitory ($R^{\ell }\to E_{-}^{\ell -1}$) synapses onto other units. However, such a violation can be mitigated by replacing computational units in SNN-PC ($R_{j}^{\ell }$, $E_{+,i}^{\ell }$, and $E_{-,i}^{\ell }$; Figure 2B) by cortical microcircuits that consist of pyramidal cells and interneurons (e.g., green triangle and orange circle wrapped inside purple, blue, and red contours, respectively; Figure 2C). Note that we used one pyramidal cell and one interneuron in a microcircuit for visual presentation purposes only. Using this microcircuit, we predict that an excitatory synapse between two microcircuits (e.g., $R_{m}^{\ell }$ and $E_{+,n}^{\ell }$) is formed between their pyramidal cells, whereas an inhibitory synapse between the two microcircuits consists of an excitatory connection from pyramidal cells in a microcircuit (e.g., $R_{m}^{\ell }$) to interneurons in another microcircuit (e.g., $E_{-,n}^{\ell }$), which then inhibits pyramidal cells in the same microcircuit (e.g., $E_{-,n}^{\ell }$). Future research will have to show how PC can be implemented using known anatomical connections (Douglas and Martin, 1991), laminar organization (Bastos et al., 2012; Pennartz et al., 2019), and different cell types (e.g., pyramidal, SST, VIP, and PV) (Keller and Mrsic-Flogel, 2018; O'Toole et al., 2023).

Besides offering a biologically plausible solution to accommodate binary spiking dynamics, the explicit separation of error units into positive and negative elements may also be beneficial for the algorithmic and computational efficiency of neuromorphic hardware. It only requires a straightforward subtraction between input and prediction, whereas bi-directional error units (such as postulated in reinforcement learning models based on prediction error coding by mesencephalic dopamine neurons) (Schultz et al., 1997) must first compute the difference and then compare it against a baseline firing rate to determine the sign of the error. With a certain baseline firing rate, positive and negative errors can be encoded by the range above and below it, respectively. However, for a full coverage of prediction error ranges, bidirectional error units must maintain a high baseline firing rate, thereby leading to a higher energy cost. The neuron targeted by a bidirectional error unit would also have to be equipped with a mechanism to discount for the baseline firing rate to obtain the true prediction error.

### 4.3 Feedforward gist

Another important novel feature of SNN-PC is the FFG pathway, which combines the fast feedforward sweep and the slow recurrent PC to account for a more comprehensive picture of visual processing. PC reconciles bottom-up and top-down accounts of perception by casting it as an inferential process that involves hierarchical recurrent interactions. However, the inference process requires multiple loops of recurrent processing to converge on an accurate representation of incoming sensory input. The expected latency of visual responses arising from recurrent predictive processing is not in accordance with the rapid forward spread of object- and context-sensitive neuronal activity across the visual cortical hierarchy within 100 ms of stimulus onset (Lamme and Roelfsema, 2000). While the precise contributions of feedforward and recurrent processes to perception are yet to be determined (Kreiman and Serre, 2020), we aimed to combine these two temporally distinct processes by integrating the FFG pathway in a PC architecture and asked whether the FFG pathway can improve network performance.

Reflecting on the temporal dichotomy of the two visual processes, the FFG pathway quickly establishes a high-level, coarse representation of input signals (e.g., the gist of a scene or object) and feeds it to each area of the PC hierarchy to aid the recurrent processing that slowly refines the representation. Instead of starting the iterative process of prediction error minimization from zero or arbitrary activity in representation units, the gist-like latent representation of incoming sensory inputs offers a biologically plausible starting point for predictive coding. This suggests a novel function of the feedforward activity relative to the classic hypothesis of rapid image recognition (Thorpe et al., 1996).

When we tested the impact of the FFG pathway on the learning of two-dimensional visual images, our results showed that its presence during training leads to reduced consistency between internal representations in higher areas with input image statistics (Figure 8). Despite the faithful reconstruction of novel and perturbed sensory inputs, which is largely driven by the recurrent dynamics of the prediction error minimization loop, the diminished classification accuracy in the absence of FFG suggests that the latent representations formed without gist inputs have extracted less information from the image statistics (Figure 8D). These findings suggest that the FFG plays a modest, but statistically significant role, in achieving classifiable latent representations by placing an a priori constraint on the inference process. Effectively, the FFG can be said to install a prior in the network based on which the PC machinery refines the representation, and which improves classifiability.

Meanwhile, it is not clear whether the same populations of cortical neurons may be involved in both the feedforward sweep and recurrent PC. Instead of performing a series of feedforward feature extraction and integration steps to elicit object-sensitive responses in high visual areas, feedforward connections in the PC network convey prediction errors. Therefore, the two processes might take separate routes. For instance, recurrent processes governed by PC may occur via cortico-cortical pathways, whereas the feedforward sweep may be mediated by the pulvino-cortical pathway or by bottom-up projections from low-level visual areas like lateral occipital cortex (Vinberg and Grill-Spector, 2008; Jaramillo et al., 2019). Such involvement of subcortical pathways is in line with the brain not being strictly hierarchical (Suzuki et al., 2023). Alternatively, the FFG pathway can be explained as a combination of the fast feedforward sweep and subsequent top-down modulation: an instantaneous feedforward sweep may activate gist units, conceptualized as IT, PFC, or other high-level cells; this may then be followed by top-down projection of activity from gist to representation units in lower visual areas (e.g., V1, V2, V3 and V4).?

In both scenarios, we assume innate and non-plastic feedforward connections in the FFG pathway. A recent study (Tschantz et al., 2023) examined how such feedforward connections can be trained via amortized inference and also showed robust perceptual capacity with shorter error convergence time and fewer training samples. However, we note that SNN-PC is spike-based and unsupervised, whereas the model in Tschantz et al. (2023) is rate-based and uses a mix of supervised and unsupervised learning.

### 4.4 Limitations on scalability

While our results show that SNN-PC can generalize what it had learned from a training set to novel instances of the test set (Figure 7; Clean), higher areas (area 2 and 3) were excluded from analyses beyond Figure 6 due to their weak correlations to input images and subpar reconstruction performance, implying that their representations in the latent space do not capture the underlying statistics well. Despite the decreasing prediction error in higher areas in the PC hierarchy during training (Figure 6B; areas 2 and 3), the progressive decrease in representational similarity across the hierarchy (Figure 6C; areas 2 and 3) suggests a loss of information about the inputs. A possible source of information leakage is the spiking mechanism of a neuron, which fires upon reaching a threshold membrane potential (*V*_θ_), that renders the input-output curve of a current-based spiking neuron non-linear. By consequence, a signal (e.g., an input current of 1,000 pA) loses its strength as it propagates through a series of neurons. Given that each cortical processing area in SNN-PC can reliably reconstruct inputs from the lower network area (Figure 6A) and that Area 1 generates internal representations of input images with a high decodability (Figures 7D, 8D), the most likely location for the information leak would be between representation and error neurons within the same area ($R^{\ell }\to E_{+/-}^{\ell }$). Addressing this leakage will require further work in future studies.

Again, we want to stress that the classification capacity of learned representations is only a byproduct of our original goal: learning a generative model. We also emphasize that, despite the low number of training samples, and the adjustments made to the PC algorithms that facilitate spike communication, we demonstrate generative capacity via reconstruction of previously unseen images (Figure 7A; Clean) and robustness against noise and occlusion (Figure 7A; Noise and Occlude). However, as for classification results, the decoding accuracy did not increase when ascending hierarchical processing areas. This is of no surprise, given the sole objective function of local prediction error minimization: there is no constraint during the training phase to learn to categorize inputs; the network is only instructed to learn internal representations that can reconstruct inputs from the area immediately below. In fact, SNN-PC fulfills this objective (Figures 6A, B). Meanwhile, we think that SNN-PC can be converted to a competitive discriminative model, if the topmost area would be clamped to class labels corresponding to inputs to the lowest area during training to learn class-specific representations via supervised learning (as in Whittington and Bogacz, 2017). This approach, however, would also obviously compromise the pursuit of biological realism.

### 4.5 Future directions

There are various other ways in which future studies may extend the biological details and/or perceptual capacities of SNN-PC. To name a few, first, a point spiking neuron can be replaced by a cortical microcircuit with multiple interneurons (e.g., Figure 2C) and with a columnar organization to replicate experimental findings and make predictions for new experiments. Second, spiking neuron behavior or connectivity can be altered to implement receptive fields and response properties to construct an invariant object representation (c.f. Brucklacher et al., 2023). Third, self-recurrent loops or online learning rules such as STDP may be employed to deal with a continuous stream of sensory inputs. Fourth, a population coding regime can be implemented to improve the reliability of signal transmission (Boerlin and Denève, 2011). Fifth, a different sensory modality can be added to perform multi-sensory integration, following the rate-based predecessor of our current SNN-PC model (Dora et al., 2021), which has been implemented in a rodent robot that performs bimodal integration of vision and touch to navigate in a maze (Pearson et al., 2021). Sixth, the network can be scaled up to better reflect the areas involved in the visual processing hierarchy. Seventh, while requiring novel learning rules, different coding schemes known to exist in the brain, such as temporal coding (Konishi, 2000; Van Rullen and Thorpe, 2001; Ono and Oliver, 2014), phase coding (O'Keefe and Recce, 1993), and the use of neural synchrony (Gray et al., 1989; Singer, 1999; Knoblauch and Palm, 2001; Brette, 2012), can be explored to make use of computational advantages offered by spiking signals.

## 5 Conclusion

We have described how to build a PC model of visual perception using biologically plausible components such as spiking neurons, Hebbian learning, and a FFG pathway. As one of the first purely spike-based and completely unsupervised PC models of visual perception, SNN-PC successfully performs perceptual inference and learning as shown by reconstruction of MNIST digit images. Also, it can denoise and show robust decodability of class information from noisy and partially occluded images. Our findings may inspire machine learning, neuromorphics, neuroscience and cognitive science communities to seek avenues moving closer to mimic the nature's most intelligent and efficient system, the brain.

## Data availability statement

The original contributions presented in the study are included in the article/supplementary material, further inquiries can be directed to the corresponding author.

## Author contributions

KL: Writing – review & editing, Writing – original draft, Visualization, Software, Methodology, Investigation, Formal analysis, Conceptualization. SD: Writing – review & editing, Methodology, Investigation, Conceptualization. JM: Writing – review & editing, Methodology, Conceptualization. SB: Writing – review & editing, Supervision, Methodology, Investigation, Conceptualization. CP: Writing – review & editing, Supervision, Resources, Methodology, Investigation, Funding acquisition, Conceptualization.
